# First Identification of “*Brachyspira hampsonii*” in Wild European Waterfowl

**DOI:** 10.1371/journal.pone.0082626

**Published:** 2013-12-04

**Authors:** Francisco Javier Martínez-Lobo, Álvaro Hidalgo, Marta García, Héctor Argüello, Germán Naharro, Ana Carvajal, Pedro Rubio

**Affiliations:** Infectious Diseases and Epidemiology Unit, Faculty of Veterinary Medicine, University of León, León, Spain; University of Maryland, United States of America

## Abstract

*Anseriformes* deserve special attention in the epidemiology of *Brachyspira* spp. because diverse *Anseriformes* species have been described to act as highly efficient carriers of several *Brachyspira* spp. that can also infect livestock. The aim of this study was to investigate the prevalence and diversity of *Brachyspira* spp. in waterfowl that winter in Spain. *Brachyspira* spp. were isolated from 51 of the 205 faecal samples collected from graylag geese and mallards in the Villafáfila Lagoons Nature Reserve (Northwestern Spain). The *Brachyspira* species identified through phenotyping, PCR and sequencing of the *nox* gene were *B. pilosicoli* (5.9%), *B. alvinipulli* (11.8%), "*B. hampsonii*" (19.6%), *B. murdochii* (23.5%) and *B. innocens* (39.2%). The most relevant finding of this study is the description of "*B. hampsonii*" in specimens from birds for the first time. Phylogenetic analysis of the *nox* gene sequences grouped all of the obtained "*B. hampsonii*" isolates into a cluster with *Brachyspira* strains previously identified by others as "*B. hampsonii*" and separated from other *Brachyspira* spp. isolates and reference strains. Additionally, this cluster was related to clades that grouped *B. murdochii* and *B. innocens* isolates. The identification of “*B. hampsonii*” was also achieved in 8 of the 10 isolates by sequencing the16S rRNA gene and *tlyA* gene. Regardless of the species identified, no antimicrobial resistance was observed in any of the enteropathogenic isolates recovered. This is the first description of “*B. hampsonii*” in European waterfowl, which might represent hosts that serve as natural reservoirs of this *Brachyspira* species. This finding indicates that this spirochete is not limited to North America, and its presence in wild birds in Europe poses a risk of transmission to livestock.

## Introduction

*Spirochaetes* are frequently present in the gastrointestinal tracts of mammals, including human beings, and some birds. The genus *Brachyspira* belongs to this phylum and comprises Gram negative, helically coiled, highly motile and anaerobic bacteria. A total of seven species have been assigned to this genus (i.e., *B. aalborgi, B. alvinipulli, B. hyodysenteriae, B. innocens, B. intermedia, B. murdochii and B. pilosicoli*), and several other species have been officially proposed, although they are not yet fully recognised as members of this genus (i.e., ‘‘*B. canis*’’, “*B. suanatina*”, “*B. rattus*”, “*B. pulli*”, “*B. ibaraki*”, “*B. corvi*”, “*B. muri*” and “*B. muridarum*”). Although the pathogenicity of some of these *Brachyspira* species has not been completely elucidated and their role in the development of intestinal diseases in their hosts remains uncertain, some of these species are unquestionably recognised as livestock enteropathogens. Thus, *B. alvinipulli*, *B. intermedia* and *B. pilosicoli* are considered to cause diarrhoea, reduced egg production and faecal staining of eggshells in chickens [1]. Additionally, *B. pilosicoli* causes spirochaetal diarrhoea in swine and has been associated with diarrhoea in a variety of mammalian hosts, such as humans, non-human primates, dogs and horses [2–4].

However, the most important disease caused by *Brachyspira* spp. is swine dysentery (SD), a severe mucohaemorragic colitis found in swine. This condition is caused by the strongly β-haemolytic spirochaete *B. hyodysenteriae* and is characterised by moderate-to-high mortality rates, poor feed conversion, decreased growth rates and a high cost of antibiotic treatments. Due to its high economic impact, SD is considered one of the most significant diseases of swine worldwide. Recent studies have shown that other proposed strongly β-haemolytic *Brachyspira* species are able to induce a clinical disease that is indistinguishable from that caused by *B. hyodysenteriae* following the inoculation of growing pigs [5,6]. For example, pigs inoculated with “*B. suanatina*” obtained from mallards exhibited diarrhoea and colitis similar to those observed in pigs affected by SD [7]. More recently, strongly β-haemolytic isolates of *Brachsypira* spp. have been isolated from pigs from North America showing clinical cases of mucohaemorragic diarrhoea [6,8], but these isolates have not been identified as *B. hyodysenteriae* through the usual PCR diagnostic tests. The experimental inoculation of these isolates into growing pigs caused mucohaemorrhagic diarrhoea and colitis that was indistinguishable from SD [6,9]. Phylogenetic analysis of the 16s rRNA and *nox* genes revealed that these isolates belong to a new species that is virulent for swine, provisionally designated “*B. hampsonii*”, which is circulating among the pig populations of the USA and Canada. However, the origin and/or non-porcine reservoirs of this novel species are entirely unknown. 

Regarding the epidemiology of *Brachyspira* spp. in livestock, *Anseriformes* deserves special attention. For example, wild-living mallards have been described as highly efficient carriers of several *Brachyspira* species that are normally found in livestock [10]. Similarly, waterfowl are thought to play a significant role in the faecal contamination of drinking water sources and agricultural crops and may also come into close contact with livestock in outdoor herds [11], thereby allowing any *Brachyspira* spp. they carry to come into contact with different potential hosts. Furthermore, migratory *Anseriformes*, travelling from breeding to feeding and resting areas and vice versa, might carry *Brachyspira* spp. over long distances and act as a potential source of pathogenic isolates for livestock in different countries. In this regard, in Europe, thousands of graylag geese (*Anser anser*) and other migrating waterfowl travel from summer breeding areas in Northwestern Europe to lagoons in the south, particularly in Spain, where they spend the winter. Most of these waterfowl populations migrate along the Atlantic flyway, which passes through several pig-producing areas of Western Europe. Additionally, *Brachyspira* spp. of avian origin might harbour antimicrobial resistance that could be transferred to livestock and make the control of *Brachyspira* spp. in domestic populations more difficult. Although information on the antimicrobial susceptibility of *Brachyspira* spp. of avian origin is scarce, decreased susceptibility to β-lactams has been described in *B. pilosicoli* isolates obtained from wild-living mallards [12]. This information points towards the necessity of testing the antimicrobial susceptibility of enteropathogenic *Brachyspira* spp. isolates from waterfowl to determine the occurrence of antimicrobial resistance in these populations and evaluate the risk of transmission to livestock populations.

The aim of this study was to investigate the prevalence, diversity and antimicrobial susceptibility of *Brachyspira* spp. isolates from waterfowl that winter in Spain, focusing primarily on enteropathogenic species found in swine, such as *B. pilosicoli*, *B. hyodysenteriae*, “*B. suanatina*” and the recently described “*B. hampsonii*”.

## Materials and Methods

### Ethics statement

No animal was disturbed, captured or manipulated in any way in carrying out this study, and faeces were collected from the ground only after the birds had left the area by choice. All sampling procedures were authorised by the Villafáfila Lagoons Nature Reserve Director. No ethical authorisation from an Animal Experimental Committee was needed because no animals were handle during sampling, according to European legislation (Directive 2010/63/EU).

### Geographical study area, wild bird population and sampling procedure

**Figure 1:**
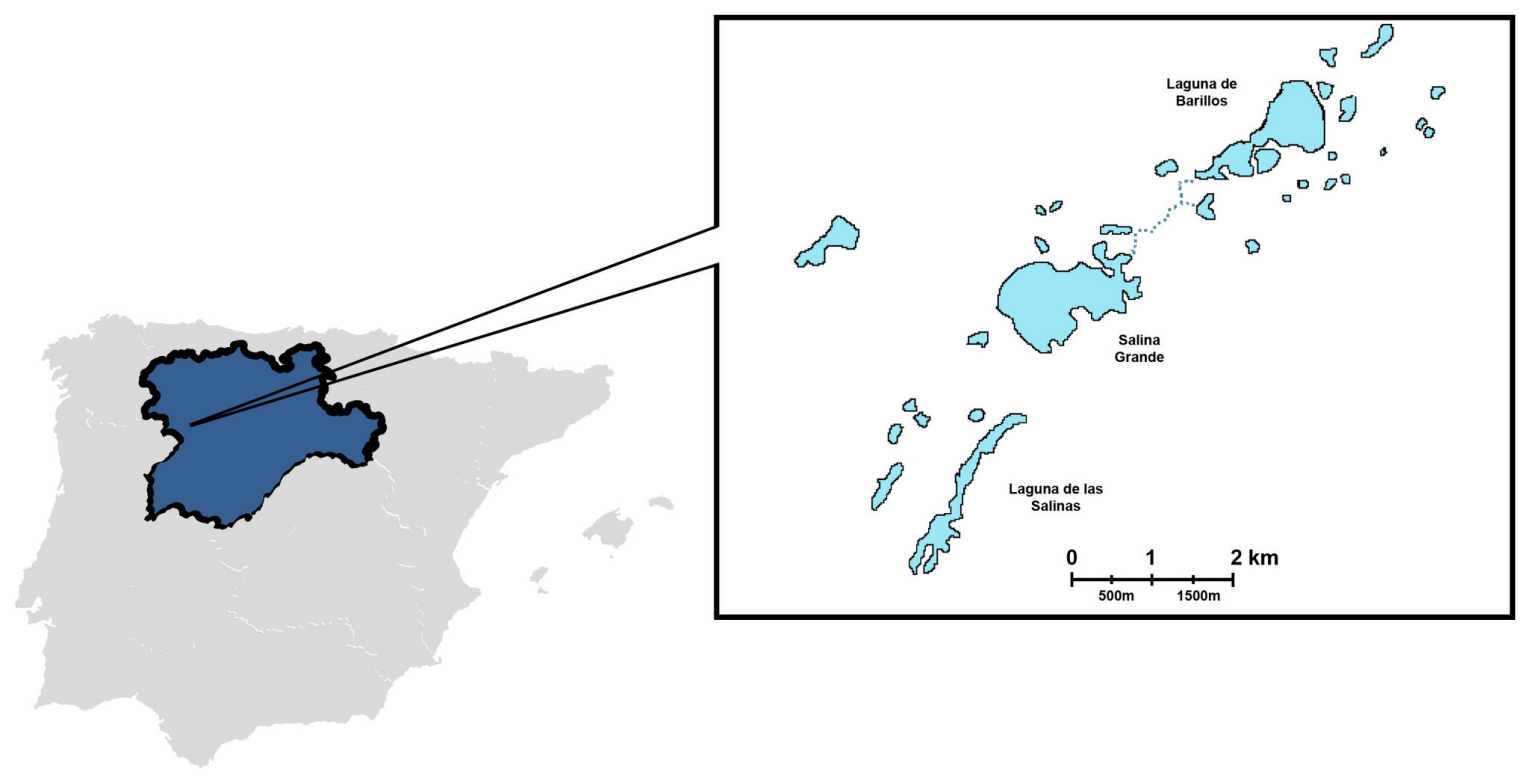
Location and topology of the Villafáfila Lagoons Nature Reserve.

Villafáfila Lagoons Nature Reserve is located on the grain-producing plains of Zamora province in the Castilla y León region (Spain). The Reserve covers a protected area of 32,682 ha and includes a complex of temporary saline lagoons that occupy approximately 600 ha (approx. 41°49’51’’ N; 5°36’31’’ W) in the highest flood years. The three main lagoons cover areas of 70 to 192 ha during the winter and lose most of their flooded area during the summer (Figure 1). The Nature Reserve was established to protect one of the world’s densest populations of great bustards (*Otis tarda*), with more than 3.000 birds inhabiting the area. The Reserve serves as a wintering area mainly for waterfowl coming from Northern European countries. Its populations of geese, particularly the graylag goose (*Anser anser*), are especially numerous, reaching sizes of up to 40.000 birds, while much smaller populations of other species, such as the greater white-fronted goose (*Anser albifrons*), are observed. The Reserve’s duck populations are also remarkably high during winter, ranging from 3.000 to 14.000 birds. There are many species of ducks, but the most common are mallards (*Anas platyrhynchos*) and northern shovelers (*Anas clypeata*). Other migratory birds that winter in Southern Spain, such as the common crane (*Grus grus*), use the lagoon complex as a resting area during their migrations.

On 24 November 2010 and 31 January 2011, a total of 205 freshly deposited waterfowl faeces specimens were randomly sampled from different locations around the lagoons. During sampling, areas where only one species usually rested were selected, and species identification was performed by Reserve Rangers. When the birds left the area, the team entered the area, and all faeces were analysed by an ornithologist to determine the species of origin. Individual swabs were taken from fresh faecal samples found on the ground. All of the swabs were immediately immersed in Amies medium (sodium chloride 3.00 g/L; potassium chloride 0.20 g/L; calcium chloride 0.10 g/L; magnesium chloride 0.10 g/L; monopotassium phosphate 0.20 g/L; disodium phosphate 1.15 g/L; sodium thioglycollate 1.00 g/L; charcoal 10.00 g/L; bacteriological Agar 7.50 g/L) and submitted to the laboratory.

### Spirochete isolation

The swabs were refrigerated and immediately transported to the laboratory. Samples were selectively cultured anaerobically on tryptose soy agar medium supplemented with 5% ovine blood and antibiotics, as previously described by Jenkinson and Wigar [13]. The cultures showing haemolytic areas within 7 days of incubation were checked for the presence of spirochaetes via phase-contrast microscopy and subsequently subcultured on fastidious anaerobe agar (FAA) plates. To remove any possible contaminating non-spirochaetal bacteria, one 5 µl loop filled with the bacteria was suspended in brain heart infusion broth (BHI), and 200 µL of the inoculum was deposited on sterile 0.45-µm pore-size filter paper, which was applied to the surface of an FAA plate. Following 15-20 minutes of incubation, the filters were removed, and the plates were incubated in an anaerobic atmosphere at 42°C for 72 h. These filtered isolates were subculture twice on FAA plates and examined for purity via phase-contrast microscopy after 72 h of incubation. Finally, all of the isolates were cloned through limiting dilution. For this purpose, 10-fold dilutions of the isolates were performed in BHI and seeded onto new FAA plates to obtain presumably pure spirochetal genotypes. The spirochaetes were stored as described elsewhere [13].

### Phenotypic characterisation

Phenotypic characterisation was performed according to a previously described panel system [13]. Under this system, 3-day-old cultures were tested for β-haemolysis on sheep blood agar and classified as strongly or weakly haemolytic. Additionally, indole production was investigated through the spot indole test, and finally, hippurate hydrolysis, α-galactosidase and β-glucosidase activity were investigated using commercial tablets (Rosco Diagnostica, Taastrup, Denmark) following the manufacturer´s instructions.

### DNA preparation and PCR

DNA was obtained from each isolate by boiling bacterial lysates. For this purpose, a loopfull of a 3-day-old culture was picked from an FAA plate and washed in phosphate-buffered saline, then boiled in nuclease-free-water and centrifuged. Next, the supernatant was transferred to a sterile microtube, and the DNA concentration was adjusted to 20 ng/µL before being used as template for PCR detection of the putative *B. hyodysenteriae* haemolysin regulatory gene *tlyA* and the *B. pilosicoli* 16S rRNA gene [15]. Furthermore, partial sequences of the *nox* and 16S rRNA genes were amplified by PCR using previously published techniques [16,17]. The *B. hyodysenteriae* B204^R^ (ATCC 31212) and *B. pilosicoli* P43/6/78^T^ (ATCC 51139) strains and nuclease-free water were employed as positive and negative controls, respectively.

### Purification and nucleotide sequencing of the PCR products

The PCR products of the partial *nox*, 16S rRNA and *tlyA* gene sequences were purified using a commercial kit (Ilustra^TM^ GFX^TM^ PCR DNA and Gel Band Purification Kit, GE Healthcare, USA), following the manufacturer’s instructions. The individual sequences of both strands of the selected PCR products were analysed via capillary electrophoresis in an automated sequencer (MegaBAce 500, GE Healthcare, USA). At least two different PCR products from each isolate were sequenced to verify that no errors had occurred during DNA amplification.

### Phylogenetic tree construction and sequence analysis

The partial *nox* gene nucleotide sequences were determined for all isolates. The sequences were aligned using Clustal W software [18] and manually edited, purged of errors and corrected. For phylogenetic comparison, distance estimation was performed for the partial *nox* gene nucleotide sequences according to the Kimura 2-parameter method [19]. A phylogenetic tree was constructed using the neighbour-joining method and including "*B. corvi*" as an outgroup. To assess the statistical reliability of the generated dendrograms, bootstrapping values were calculated (1,000 replicates). The analyses were performed using the MEGA 5.1 software [20]. PCR products for the 16S rRNA gene were only obtained for those isolates that clustered with a *Brachyspira* spp. classified by others as "*B. hampsonii*" in the phylogenetic tree based on the partial *nox* gene sequences. These 16S rRNA sequences were analysed as described above.

The positive amplicons of the partial *tlyA* gene obtained through diagnostic PCR specific for *B. hyodysenteriae* were purified and sequenced as described above. To determine whether the *Brachyspira* spp. isolates positive for the *tlyA* gene could be classified as *B. hyodysenteriae*, amino acid sequences were predicted using BioEdit 7.0 [21] and compared to sequence data deposited in the GenBank database. For comparative purposes, *Brachyspira* spp. sequences were retrieved from the GenBank database and included in the phylogenetic analysis, based on the previous nBLAST results generated in our laboratory (Table 1).

**Table 1:** The type and reference strains as well as the field isolates of *Brachyspira* spp. used for comparative purposes in the phylogenetic analysis.

			**GenBank accession no.**		
**Strain and species affiliation**	**Phenotype^1^**	**Host specie**	***nox* gene**	**16S rRNA gene**	***tlyA* gene**
“*Brachyspira hampsonii*” NSH-7	20001^2^	Pig	JX232308	JX232342	
“*Brachyspira hampsonii*” NSH-5	20001	Pig	JX232306	JX232340	
*Brachyspira* sp. KC35	20001	Pig	JX197410	JX197406	
*Brachyspira* sp. EB106	20001	Pig	JX197409	JX197405	
*Serpulina* sp. P280/1	20001	Pig	AF060815	NA	
*B. hyodysenteriae* WA1	21001	Pig	CP001357	CP001357	CP001357
*B. hyodysenteriae* AN1409:2/01	21001	Mallard	DQ487115	AY352281	
*B. hyodysenteriae* B204	21001	Pig	U19610	U14932	X61684
“*B. suanatina*” AN1418:2/01	21001	Mallard	DQ487124	AY352282	
*B. intermedia* AN519/97	11001	Pig	EF517542	EF517536	
*B. intermedia* AN983/90	11001	Pig	EF517543	U14933	
*B. intermedia PWS*	11001	Pig	AF060811	EF488166	CP002874
*B. innocens* C336	10011	Pig	EF517546	U14919	
*B. innocens* C173	10011	Pig	NA	U14921	
*B. murdochii* 56-150T	10001	Pig	AF060813	AY312492	NC_014150
*B. murdochii* KC60	10001	Pig	JX197411	NA	
*B. pilosicoli* P43/6/78T	10210	Pig	AF060807	U14927	CP002025
*B. pilosicoli* AN3590:1/1/02	10210	Mallard	JF430748	JF430708	
“*B. pulli*” AN304/04	10001	Chicken	JF430769	EF164986	
*B. alvinipulli* C1T	10101	Chicken	AF060814	EF455559	
*B. alvinipulli* AN3382/2/03	10101	Chicken	JF430770	EF164987	
*B. alvinipulli* AN33/1/02	10101	Mallard	JF430743	JF430704	JF430733
“*B. corvi*” AN968/2/04	10010	Hooded crow	EU819075	EF371462	

^1^ Phenotypic results are given in the following order: intensity of β-haemolysis, indole, hippuricase, α-galactosidase, β-glucosidase.

^2^ Results of phenotypic tests: 1= weak reaction; 2= strong reaction; 0= negative result.

### Antimicrobial susceptibility

The antibiotic susceptibility of the isolates classified as “*B. hampsonii*” in this study was determined via the minimal inhibitory concentration (MIC) method using a commercially available system (VetMIC Brachy antibiotic panels; SVA, Sweden), following the manufacturer's recommendations. VetMIC Brachy antibiotic panels consist of 48-well plates, in which two-fold serial dilutions of tiamulin (0.063 to 8 μg/mL), valnemulin (0.031 to 4 μg/mL), doxycycline (0.125 to 16 μg/mL), tylvalosin (0.25 to 32 μg/mL), lincomycin (0.5 to 64 μg/mL) and tylosin (2 to 128 μg/mL) are tested. The MIC was determined to be the lowest concentration of the antimicrobial agent that prevented visible growth of the bacteria. The absence of contamination was confirmed by phase-contrast microscopy in wells where visible growth was observed.

## Results

### Isolation and phenotypic characterisation

The number of faecal samples obtained, the bird species affiliations and the prevalence of spirochaetal faecal shedding are provided in Table 2. A total of 205 faecal swabs from four bird species were collected in this study. Most of these specimens came from graylag goose (71.7%) or wild mallards (16.5%), and only a few came from great bustard and common crane. Growth of spirochaetes exhibiting weak or strong β-haemolysis was observed in 51 samples (24.9%) from waterfowl, whereas no haemolysis and/or spirochaetal growth was observed in any of the samples from other species. Following primary isolation, almost 50% of the isolates were found to be contaminated with coccoid bacteria, as determined via phase-contrast microscopy, but the contaminants were completely removed after the filtration process. The spirochaete isolates varied microscopically in shape and motility, although no correlations were observed between the morphology of the isolates and the *Brachyspira* species or host of origin. Additionally, they displayed different growth patterns on FAA plates after 72 h of incubation, although the differences could not be ascribed to any particular *Brachyspira* species. The phenotypic profiles of the pure isolates are provided in Table 3. Most of the obtained *Brachyspira* spp. isolates belonged to biochemical groups IIIa or IIIbc, which correspond to *B. murdochii* and *B. innocens*, respectively. The remainder of the isolates showed biochemical properties corresponding to *B. alvinipulli* or "*B. hampsonii*”. In contrast, phenotypic profiles consistent with *B. hyodysenteriae*, *B. intermedia* or *B. pilosicoli*, i.e., groups I, II and IV, were not detected.

**Table 2:** Numbers of processed samples and spirochaetal prevalence in waterfowl faeces collected at the Villafáfila Lagoons Nature Reserve in the winter of 2011-2012.

				**No. of samples**	
**Common name**	**Scientific name**	**November 2011**	**January 2012**	**Total**	**No. of positive**
Graylag goose	*Anser anser*	49	98	147	34 (23.1%)
Mallard	*Anas platyrrynchos*	34	-	34	17 (50.0%)
Common crane	*Grus grus*	16	-	16	-
Great bustard	*Otis tarda*	8	-	8	-
Total		107	98	205	51 (24.9%)

**Table 3:** Phenotypic profiles of the 51 *Brachyspira* spp. isolates recovered from faecal samples from geese and mallards collected in northwestern Spain (Villafáfila Lagoons Nature Reserve).

**Biochemical Group**	**Indicated species**	**No. of isolates**	**%**	**Haemolysis**	**Indole**	**Hippurate**	**α-Galactosidase**	**β-Glucosidase**
I	*B. hyodysenteriae “B. suanatina”*	0	0	Strong	+	-	-	+
II	*B. intermedia*	0	0	Weak	+	-	-	+
IIIa	*B. innocens*	20	39.2	Weak	-	-	+	+
IIIbc	*B. murdochii*	12	23.6	Weak	-	-	-	+
IV	*B. pilosicoli*	0	0	Weak	-	+	-	-
-	*B. alvinipulli*	7	13.7	Weak	-	+	-	+
-		2	3.9	Weak	-	+	+	+
-	*“B. hampsonii”*	10	19.6	Strong	-	-	-	+

### Sequence analysis and species affiliations

**Figure 2:**
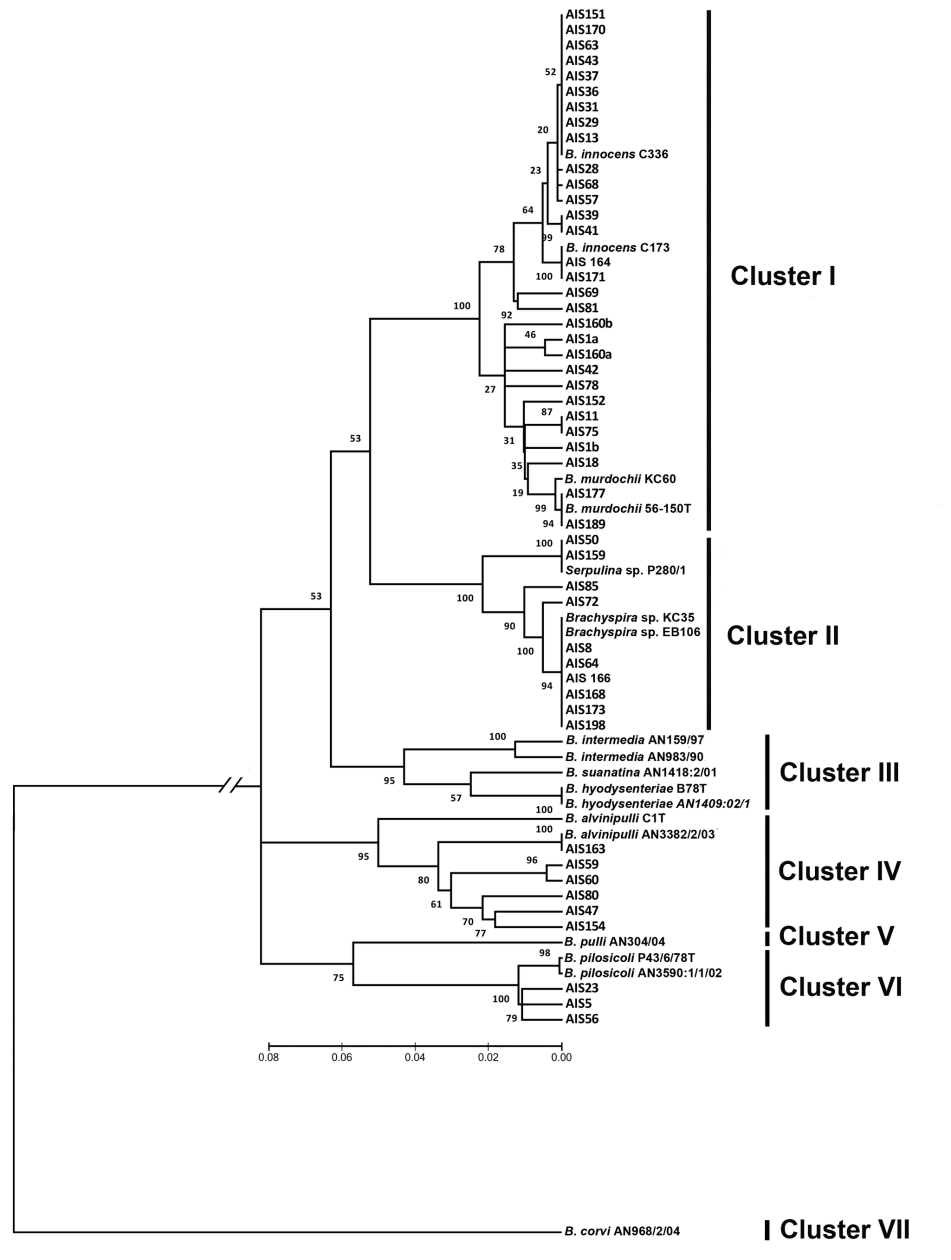
Phylogenetic tree based on *nox* gene sequences (893 nt) showing the evolutionary relationships among *Brachyspira* spp. isolates from graylag geese and mallards. The tree was constructed using the neighbour-joining method. The bar represents the distance equivalent to 10 substitutions per 100 nucleotide positions on the upper subbranch. "*B. corvi*" AN968/2/04 was used as an outgroup. The main clusters were designated I-VII.

All of the partial *nox* gene sequences analysed were 893 nt long. The phylogenetic relationships among the *Brachyspira* spp. isolates and reference strains are depicted in an evolutionary tree (Figure 2). The evolutionary tree shows seven monophyletic clusters corresponding to *B. murdochii* and *B. innocens* (I), "*B. hampsonii*” (II), *B. hyodysenteriae*, *B. intermedia* and "*B. suanatina*” (III), B. *alvinipulli* (IV), *B. pulli* (V), *B. pilosicoli* (VI) and "*B. corvi*" (VII). The isolates obtained in this study clustered with 4 of the 7 groups described. Most of the isolates belonged to cluster I, although they were allocated into two separate subclusters, harbouring isolates that are biochemically similar to *B. innocens* and to *B. murdochii* reference strains, respectively. Additionally, some isolates that were phenotypically similar to *B. alvinipulli* reference strain C1^T^ grouped in cluster IV. Finally, three isolates clustered with *B. pilosicoli* reference strains in cluster VI. Interestingly, ten isolates grouped together with *Brachyspira* strains that were recently identified as "*B. hampsonii*” in cluster II. This cluster was independent of the other *Brachyspira* spp. isolates and spirochete reference strains, although it was related to cluster I, which included *B. murdochii* and *B. innocens* isolates. Phylogenetic analysis of the nucleotide sequences of these isolates revealed that they were divided into two monophyletic clades. The first clade included 8 of the isolates obtained in this study, grouped with the previously described "*B. hampsonii*” (i.e., *Brachyspira* sp. KC35 and *Brachyspira* sp. EB106). Six of these isolates were closely related to previously described isolates, while 2 of them (isolates AIS72 and AIS85) constituted independent branches of the above-mentioned clade. Finally, the remaining two isolates (i.e., AIS 50 and AIS159) clustered with *Serpulina* sp. P280/1 in a clearly defined and separated clade of cluster II. 

The species affiliations of the 51 *Brachyspira* spp. isolates obtained in this study, based on the combination of their phenotypic profiles and phylogenetic analysis of *nox* gene sequences, as well as the GenBank accession numbers of the *nox* sequences (i.e., KF386036 to KF386086) are listed in Table 4. As can be observed in the table, most of the isolates were classified as *B. innocens* (39.2%) or *B. murdochii* (23.5%). The remaining 47.3% of the isolates were recognised as "*B. hampsonii*" (19.6%), *B. alvinipulli* (11.8%) or *B. pilosicoli* (5.9%), while the species *B. intermedia*, *B. hyodysenteriae* and "*B. suanatina*" were not identified in this study. Discrepancies between the phenotyping and genotyping results were only found in the three isolates identified as *B. pilosicoli*, with isolate AIS5 showing a phenotype that was consistent with *B. alvinipulli*, while the other 2 isolates lacked indole production and displayed hippurate cleavage and α-galactosidase and β-glucosidase activities. 

**Table 4:** Classification of the *Brachyspira* spp. isolates recovered from faecal samples from graylag geese and mallards collected in northwestern Spain (Villafáfila Lagoons Nature Reserve) based on phenotypic and molecular results.

**Isolate**	**Proposed specie**	**GenBank acc. nos.**	**Species of origin**	**Phenotype^1^**	**Isolate**	**Proposed specie**	**GenBank acc. nos.**	**Species of origin**	**Phenotype**
AIS1a	*B. innocens*	KF386036	Goose	10011^2^	AIS69	*B. innocens*	KF386062	Mallard	10011
AIS1b	*B. murdochii*	KF386037	Goose	10001	AIS72	*"B. hampsonii"*	KF386063	Mallard	20001
AIS5	*B. pilosicoli*	KF386038	Mallard	10101	AIS74	*B. murdochii*	KF386064	Mallard	10001
AIS8	*"B. hampsonii"*	KF386039	Mallard	20001	AIS75	*B. murdochii*	KF386065	Mallard	10001
AIS11	*B. murdochii*	KF386040	Goose	10001	AIS78	*B. murdochii*	KF386066	Mallard	10001
AIS13	*B. innocens*	KF386041	Goose	10011	AIS80	*B. alvinipulli*	KF386067	Mallard	10100
AIS18	*B. murdochii*	KF386042	Goose	10001	AIS81	*B. innocens*	KF386068	Mallard	10011
AIS23	*B. pilosicoli*	KF386043	Goose	10111	AIS85	*"B. hampsonii"*	KF386069	Goose	20001
AIS28	*B. innocens*	KF386044	Mallard	10011	AIS151	*B. innocens*	KF386070	Goose	10011
AIS29	*B. innocens*	KF386045	Goose	10011	AIS152	*B. murdochii*	KF386071	Goose	10001
AIS31	*B. innocens*	KF386046	Goose	10011	AIS154	*B. alvinipulli*	KF386072	Goose	10100
AIS36	*B. innocens*	KF386047	Mallard	10011	AIS159	*"B. hampsonii"*	KF386073	Goose	20001
AIS37	*B. innocens*	KF386048	Mallard	10011	AIS160a	*B. innocens*	KF386074	Goose	10011
AIS39	*B. innocens*	KF386049	Goose	10011	AIS160b	*B. murdochii*	KF386075	Goose	10001
AIS41	*B. innocens*	KF386050	Goose	10011	AIS163	*B. alvinipulli*	KF386076	Goose	10100
AIS42	*B. murdochii*	KF386051	Mallard	10001	AIS164	*B. innocens*	KF386077	Goose	10011
AIS43	*B. innocens*	KF386052	Goose	10011	AIS166	*"B. hampsonii"*	KF386078	Goose	20001
AIS47	*B. alvinipulli*	KF386053	Goose	10100	AIS168	*"B. hampsonii"*	KF386079	Goose	20001
AIS50	*"B. hampsonii"*	KF386054	Goose	20001	AIS170	*B. innocens*	KF386080	Goose	10011
AIS56	*B. pilosicoli*	KF386055	Mallard	10111	AIS172	*B. innocens*	KF386081	Goose	10011
AIS57	*B. innocens*	KF386056	Goose	10011	AIS173	*"B. hampsonii"*	KF386082	Goose	20001
AIS59	*B. alvinipulli*	KF386057	Goose	10101	AIS177	*B. murdochii*	KF386083	Goose	10001
AIS60	*B. alvinipulli*	KF386058	Goose	10100	AIS185	*B. murdochii*	KF386084	Goose	10001
AIS63	*B. innocens*	KF386059	Mallard	10011	AIS189	*B. murdochii*	KF386085	Goose	10001
AIS64	*"B. hampsonii"*	KF386060	Mallard	20001	AIS198	*"B. hampsonii"*	KF386086	Goose	20001
AIS68	*B. innocens*	KF386061	Mallard	10011	-	-	-	-	-

^1^ Phenotypic results are given in the following order: intensity of β-haemolysis, indole, hippuricase, α-galactosidase, β-glucosidase.

^2^ Results of phenotypic tests: 1= weak reaction; 2= strong reaction; 0= negative result.

Surprisingly, all of the strongly β-haemolytic isolates found in this study were phenotypically and genetically identified as "*B. hampsonii*”. The frequency of the detection of this species differed slightly depending on the origin of the samples, with only 4.9% of the goose samples being positive, while up to 9.0% of the mallard samples contained "*B. hampsonii*”. However, the proportions of "*B. hampsonii*” isolates among all of the obtained spirochaetes were very similar for the two species (20.6% for geese and 17.6% for mallards). These results are consistent with the general frequency of spirochaete isolation from the faeces samples, which was higher in mallards than in graylag goose (50.0% vs. 23.1%). Nevertheless, it is remarkable that all of the *Brachyspira* species identified in this study were found in samples from both hosts at least once, indicating that both hosts can carry various *Brachyspira* species.

**Figure 3:**
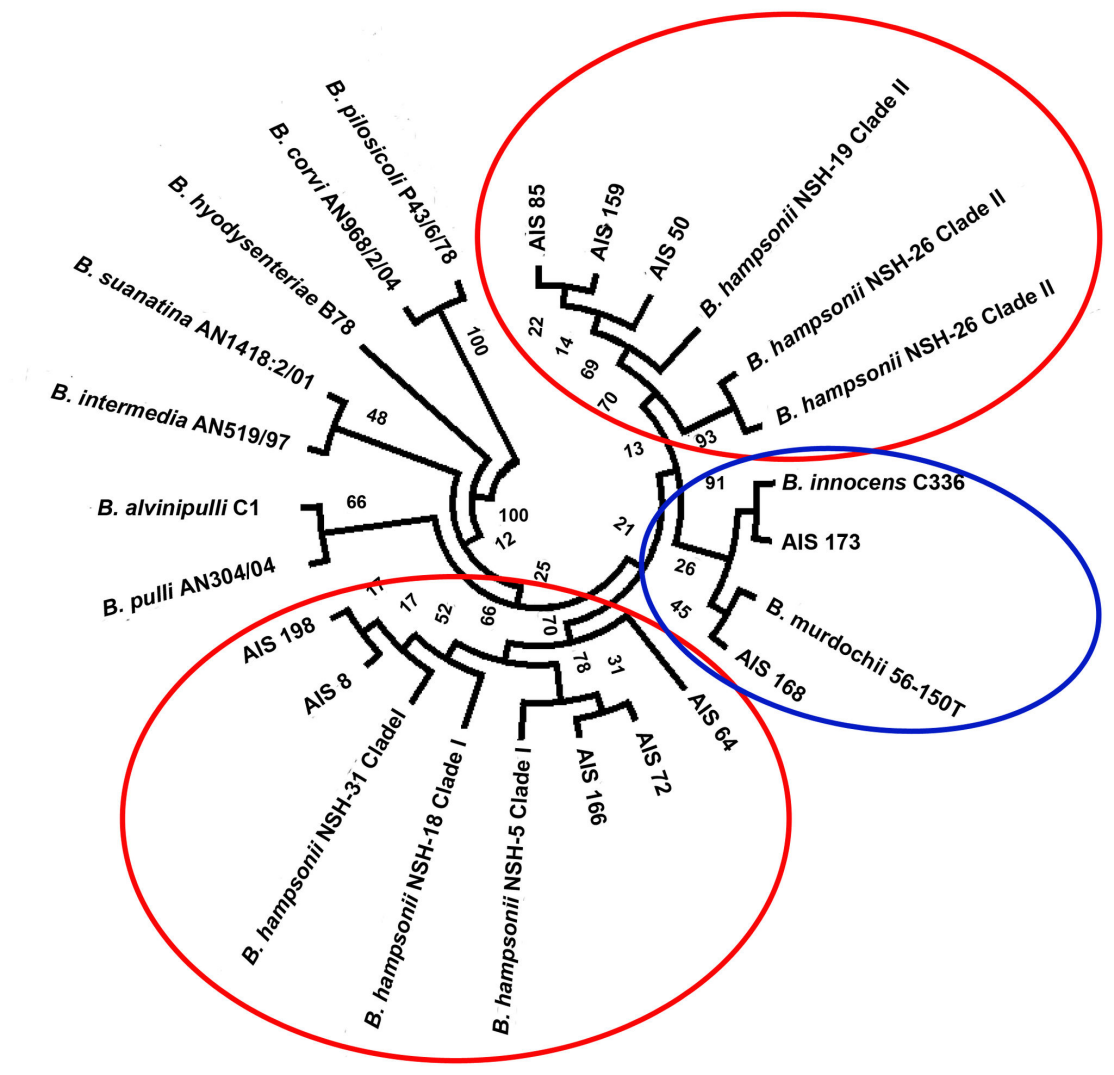
Phylogenetic tree based on 16S rRNA gene sequences (1376 nts) showing the evolutionary relationships among the obtained “*B. hampsonii*” isolates and *Brachyspira* spp. reference strains. The tree was constructed by means of the neighbour-joining method. Red circles indicate the two clusters containing the “*B. hampsonii*” reference isolates. The blue circle indicates the cluster comprising the *B. innocens* and *B. murdochii* reference strains used.

To confirm the presence of "*B. hampsonii*” in the bird fecal samples, additional gene targets were amplified and sequenced from the 10 isolates identified as "*B. hampsonii*”. For the 16S rRNA gene, 1376 nt were determined, and the sequences were used to draw a dendrogram (Figure 3). These sequences were submitted to GenBank, and accession numbers were assigned (KF386087 to KF386096). According to the phylogenetic tree defined by *nox* gene sequences, isolates AIS8, AIS64, AIS166 and AIS198 clustered with "*B. hampsonii*” clade I strains, while isolates AIS50 and AIS159 clustered with "*B. hampsonii*” clade II strains. Additionally, isolates AIS72 and AIS85, which formed two independent branches of clade I in the *nox* gene evolutionary tree, were allocated to groups formed by clade I and clade II strains, respectively, in the dendrogram derived from the sequences of the 16S rRNA gene. However, the affiliation of isolates AIS168 and AIS173 with "*B. hampsonii*” was not confirmed by the phylogenetic dendrogram based on 16S rRNA gene sequences. These isolates were rearranged and clustered with *B. murdochii* and *B. innocens* reference strains, respectively.

### Sequence analysis of amplicons from diagnostic duplex PCR

Surprisingly, positive amplicons of the *tlyA* gene of *B. hyodysenteriae* were detected in 14 isolates classified as "*B. hampsonii*” (6/14)*, B. innocens* (2/14)*, B. murdochii* (4/14) or *B. alvinipulli* (2/14), based on phenotyping and phylogenetic analysis of the *nox* gene. The *tlyA* gene sequences obtained in this study were submitted to GenBank, and accession numbers were assigned (KF386097 to KF386110).

Pairwise comparisons between the isolates indicated that AIS11, AIS18, AIS78 and AIS152, proposed as *B. murdochii*, show higher sequence similarity to *B. murdochii* reference strain 56-150T than to any other *Brachyspira* *spp.* strain, confirming their affiliation with this species. Similarly, the predicted amino acid sequences of isolates AIS63 and AIS154, which clustered with *B. alvinipulli* strain C1T in the phylogenetic tree, exhibited 100% similarity to a partial sequence from isolate AN3382/2/03 obtained from mallards in Sweden and classified as *B. alvinipulli* [10].

However, isolate AIS63, which was phenotypically and genetically consistent with *B. innocens* C336, showed a predicted amino acid sequence for the *tlyA* gene that was 99% similar to *B. murdochii* 56-150T and isolate AIS11.

Comparisons of the obtained partial *tlyA* gene sequences between "*B. hampsonii*” isolates AIS8, AIS64, AIS72, AIS85, AIS166 and AIS198 and reference strains revealed similarities ranging from 87.0% to 96.1% at the nucleotide level and from 92.9% to 97.6% at the amino acid level, depending on the species considered. Specifically, the sequences from "*B. hampsonii*” isolates showed the closest similarity to haemolysin A from *B. hyodysenteriae* WA1 (mean 97.0 ± 0.4%) and *B. intermedia* PWS (96.77 ± 0.44), followed by *B. pilosicoli* (93.78 ± 0.44), *B. alvinipulli* (93.73 ± 0.45) and *B. murdochii* (93.36 ± 0.33). 

### Antimicrobial susceptibility of “B. hampsonii” isolates

The MICs of the six antimicrobial agents tested against the “*B. hampsonii*” isolates are shown in Table 5. A clear unimodal distribution of the susceptible populations was obtained for all of the antimicrobials tested. Thus, all of the isolates were susceptible to the lowest antibiotic concentration tested, and the MICs were therefore below the lowest concentrations of the antimicrobials included in the panel.

**Table 5:** MICs (µg/mL) of six antimicrobial agents for proposed "*B. hampsonii*" isolates recovered from faecal samples from graylag geese and mallards collected in northwestern Spain (Villafáfila Lagoons Nature Reserve).

	**MIC (µg/mL)**					
**Isolates**	**Tiamulin**	**Valnemulin**	**Doxycicline**	**Tilvalosin**	**Lincomicin**	**Tylosin**
AIS8	<0.063	<0.031	<0.125	0.5	<0.5	4
AIS50	<0.063	<0.031	0.25	1	<0.5	4
AIS64	<0.063	<0.031	<0.125	0.5	<0.5	<2
AIS72	<0.063	<0.031	<0.125	0.5	<0.5	4
AIS85	<0.063	<0.031	<0.125	0.5	<0.5	4
AIS159	<0.063	<0.031	<0.125	0.5	<0.5	<2
AIS163	<0.063	<0.031	<0.125	0.5	<0.5	<2
AIS166	<0.063	<0.031	<0.125	0.5	<0.5	4
AIS173	<0.063	<0.031	<0.125	0.5	<0.5	4
AIS198	<0.063	<0.031	<0.125	1	<0.5	4

## Discussion

A number of studies have focused on estimation of the prevalence and diversity of intestinal spirochaetes in different bird populations [10,22–27] because they could represent important reservoirs of *Brachyspira* species, including those that can infect and cause disease in livestock. Together, the results of these studies show that *Brachyspira* species are commonly found in the intestines of waterfowl species. Biologically, waterfowl species exhibit prominent migratory behaviour [28]. They usually breed in a widely dispersed pattern, over large geographic areas and concentrate during winter in a few key areas [29]. Spain is one of the main wintering areas for Northern European waterfowl populations, which are distributed among the main wetlands in the country. These high concentrations of waterfowl might facilitate the transmission of spirochaetes among different avian species and from birds to livestock. Special attention should be given to the possibility of the transmission of pathogenic *Brachyspira* spp. to swine because Spain is the second largest pig-producing country in Europe, with an output of more than 41 million pigs per year. Moreover, outdoor production, particularly associated with the semi-intensive or extensive systems where Iberian pigs are reared, might facilitate contact between reservoirs of *Brachyspira* spp., including several bird species, and pigs. Therefore, the aim of this study was to investigate the prevalence of *Brachyspira* spp. species, focusing in particular on spirochaetes that are pathogenic for swine, in the bird populations of the Villafáfila Lagoons Nature Reserve, which is one of the main wintering sites for waterfowl on the Iberian Peninsula.

For this purpose, we collected faeces from different avian species and analysed these specimens for the presence of spirochaetes. Although we cannot completely rule out the possibility that faeces were mistakenly collected from other species, the likelihood of misidentification was low because the sampling areas were selected based on specific resting or feeding areas for a single species (i.e., grain plains for great bustards, crop fields for graylag geese and the banks of lagoons for mallards and common cranes). In addition, only fresh faeces were collected, increasing the probability that the faeces belonged to birds that had just left the area. Finally, the sizes of the faeces produced are quite different between the sampled species, facilitating their identification. However, molecular tools, such as DNA barcoding [30], would be necessary to achieve definitive and unambiguous identification of the species of origin for the faecal samples.

Nevertheless, our results are consistent with previous data, further indicating that the different avian species targeted were correctly identified. Thus, although a number of intestinal spirochaetes are able to colonise wild-living non-anseriform birds, such as game birds, jackdaws and rheas [26,27], *Anseriformes* have been suggested to represent some of the main carriers of *Brachyspira* spp. In accordance with this notion, spirochetes were not observed in specimens from the common crane or great bustard in this study, while several *Brachyspira* species were isolated from both graylag geese and mallards. Wild-living mallards have been reported to support a high biodiversity of *Brachyspira* spp. in their intestines, frequently excreting spirochaetes in their faeces without showing any clinical signs [10]. In contrast, disease associated with spirochaetal infection has been reported in geese. In particular, *B. alvinipulli* has been isolated from geese suffering from fibrinonecrotic typhlocolitis [31]. The results of the present study confirm that wild-living mallards can carry a variety of *Brachyspira* species and point to graylag geese as a relevant host for most of the intestinal *Brachyspira* spp. (including *B. murdochii*, “*B. hampsonii*”, *B. innocens*, *B. pilosicoli* and *B. alvinipullli*). This role has been confirmed in mallards, which have been reported to carry *Brachyspira* spp. isolates whose *nox* gene sequences are similar to those of isolates recovered from livestock [10].

Nevertheless, most of the *Brachyspira* spp. isolates obtained from waterfowl in this study belonged to non-pathogenic species or species of unknown virulence, such as *B. murdochii* and *B. innocens*, which have been reported to constitute part of the microbiota of the intestinal tract of birds and do not induce clinical disease or lesions following experimental infections [32].

Regarding the pathogenic spirochaetes recovered, *B. pilosicoli* was only identified in 2 of the 34 mallard samples and 1 of the 147 goose samples. The low frequency of isolation of this *Brachyspira* species was unexpected, especially because its reported prevalences in water bird species in Australia and wild-living mallards in Sweden are 40% and 47%, respectively [10,24]. The ultimate reasons for these differences in the frequency of detection of *B. pilosicoli* in waterfowl between our study and previous studies remain unclear. However, it is noteworthy that the prevalence reported for *B. pilosicoli* in pigs in Spain is also very low [33], and the isolation of this *Brachyspira* species from clinical samples from swine submitted to the Diagnostic Laboratory of the Department of Infectious Diseases of the Veterinary Faculty of the University of Leon, Spain, is an infrequent event. Together, these data indicate that *B. pilosicoli* might not be a common spirochete in the environment in Spain. It should be noted that none of the isolates described as *B. pilosicoli* in the present study showed the typical phenotypic profile of biochemical group IV. However, this finding was not surprising because phenotypic diversity is a common finding among *B. pilosicoli* isolates from birds and pigs [10,34], and classification must be performed via PCR, multilocus enzyme electrophoresis and/or gene sequencing.

A total of 10 strongly β-haemolytic *Brachyspira* spp. isolates were obtained from mallards and geese. Unexpectedly, none of these spirochaetes produced indole. Furthermore, the genomic analyses indicated that none of them clustered with any of the reference strains of *B. hyodysenteriae* and “*B. suanatina*” included in the study. Although "*B. suanatina*” has been isolated from mallards and pigs in Sweden and Denmark [35], no isolates of this newly proposed species have been identified among *Brachyspira* spp. collected in Germany [36]. There are also no previous reports concerning the presence of this species in Spain, and to our knowledge, it has never been isolated from pigs or birds. In the case of *B. hyodysenteriae*, previous studies carried out in Sweden have indicated that up to 20% and 10% of the spirochaetes isolated from farmed and wild-living mallards, respectively, show a phenotypic profile similar to *B. hyodysenterie* [3]. However, no information related to the distribution of this species in waterfowl in other geographic regions is available. Thus, it is possible that this *Brachyspira* species is not as frequent in waterfowl as inferred from the available studies from other areas. Nevertheless, this spirochete is commonly found in pigs in various countries, including Spain [33,36].

Surprisingly, all of the strongly β-haemolytic *Brachyspira* isolates recovered from faecal samples from waterfowl species were classified as “*B. hampsonii*” through phenotyping and genotyping. The genotypic characterisation was initially based on partial sequences of the *nox* gene. Based on these sequences, the isolates identified as “*B. hampsonii*” were clustered into two previously described clades [8]. Clade I was composed of 8 of the 10 isolates analysed. These isolates were more diverse than the two isolates grouped in clade II with *Serpulina* sp. P/280. It is remarkable that the isolates recovered from waterfowl were divided into the same two clades previously described for swine "*B. hampsonii*" isolates and that the genetic distance of the isolates included in clade I was greater for both the swine and waterfowl isolates compared to the diversity of the isolates of both origins included in clade II.

However, the analysis of 16S rRNA gene sequences resulted in rearrangement of the phylogenetic tree. Thus, 2 isolates previously considered to be “*B. hampsonii*” clustered with *B. innocens* and *B. murdochii*. The lack of correlation between these two phylogenetic dendrograms could be due to the genomic plasticity of spirochaetes. Thus, genomic rearrangements and recombination events are possible between different species of spirochaetes due to horizontal gene transfer promoted by a chromosomal prophage [37]. These events are facilitated by the fact that most *Brachyspira* spp. are commensal organisms found in the intestinal tracts of waterfowl species [7,10] and can infect the same individual concurrently [9]. For example, Jansson et al. [10] suggested that recombination had occurred in the *nox* and 16S rRNA genes of three *B. pilosicoli* isolates obtained from a single sample from a mallard. The intestinal tracts of waterfowl might represent a highly suitable habitat for recombination between spirochaetes, and these events greatly complicate the classification of isolates belonging to the *Brachyspira* genus, especially in avian hosts and/or new environmental niches. Nevertheless, based on phenotypic characterisation, all of the *Brachyspira* spp. isolates obtained in this study that were grouped together with “*B. hampsonii*” clade I or clade II based on *nox* gene sequences are proposed as “*B. hampsonii*” in this paper.

It is remarkable that the present study is the first to report the isolation of the newly proposed *Brachyspira* species “*B. hampsonii*” from wild mallards and graylag geese in Europe. Furthermore, although the sample size was limited, this newly proposed *Brachyspira* species was isolated at a relatively high frequency from waterfowl wintering in Northwestern Spain, showing an estimated prevalence close to 20%. Prior to this work, the presence of “B*. hampsonii*” was believed to be restricted to North America, where veterinary diagnostic laboratories have reported circulation of the bacteria in pig populations in Canada and the USA [5,7,8]. The presence of “*B. hampsonii*” in wild birds in Europe observed in this study might be explained by the intercontinental transmission of this species between American and European waterfowl populations because the global migratory pathways of waterfowl overlap between Europe and eastern North America, which facilitates pathogen exchange between the two populations [38,39]. Additionally, “*B. hampsonii*” has been recently isolated from lesser snow geese (*Chen caerulescens caerulescens*) in the Canadian arctic [40]. However, the origin of “*B. hampsonii*” in the European region and its relationship with North American isolates is still unknown.

Regardless of its origin, the circulation of “*B. hampsonii*” in European graylag geese and mallards poses a risk for swine because this pathogenic spirochete could theoretically spread from wild waterfowl to pigs. Thus, the relatively high frequency of the isolation of ”*B. hampsonii*” from goose faecal samples points towards a potential role of graylag geese in the epidemiology of infections by this *Brachyspira* spp. in domestic populations. However, the isolation of this species from mallards deserves special attention because, unlike the graylag goose, which is found in specific wetlands, mallards are widely distributed across a broad range of habitats, and they can be frequently observed at shallow water depths in the field. This particular behaviour might facilitate contact between contaminated water and/or crops and pigs reared in outdoor units, thereby enabling the transmission of “*B. hampsonii*” to the swine population. 

Consequently, the results of this study highlight the importance of carrying out studies examining the prevalence of “*B. hampsonii*” in the pig populations of Spain and Europe. Furthermore, our results indicate that cross-reactions can occur between *B. hyodysenteriae* and some isolates of “*B. hampsonii*” when a PCR assay designed for the routine diagnosis of swine dysentery is applied [14]. Cross-reactions between *B. hyodysenteriae* and other spirochaetes in species-specific PCR assays have been described in the past, though only for isolates phenotypically similar to *B. intermedia*, *B. innocens* and *B. alvinipulli* [10]. However, “*B. hampsonii*” can exhibit strong β-haemolysis in culture, similar to that produced by *B. hyodysenteriae*. Additionally, consistent with their similar culture properties [6], some strains of “*B. hampsonii*” have been described to be as virulent as a reference strain of *B. hyodysenteriae* [9] and to cause severe mucohaemorragic diarrhoea that is undistinguishable from swine dysentery. In fact, it has been reported that in North America, strongly β-haemolytic spirochaetes classified as “*B. hampsonii*” are more frequently isolated in cases of dysentery than *B. hyodysenteriae* [9]. Hence, the existence of cross-reactions between “*B. hampsonii*” and *B. hyodysenteriae* in a theoretically *B. hyodysenteriae*-specific PCR assay could have important consequences for the appropriate diagnosis of brachyspiral colitis in swine and for evaluation of the real incidence of “*B. hampsonii*” in the European pig population. In this regard, de Jong et al. [41] recently described a β-strongly haemolytic isolate in Europe that was positive for the *tlyA* gene but was identified as *B. intermedia* based on *nox* gene sequencing, suggesting an affiliation with “*B. hampsonii*”. All of these data indicate that routine diagnosis based on commonly used PCR assays targeting the *tlyA* gene might be misleading in this complex genomic context and that caution should be employed in the molecular identification of *Brachyspira* spp. Other PCR assays, such as those based on the *nox* and 23S rRNA genes, may be more specific for *B. hyodysenteriae* identification. However, further studies are needed to assess these diagnostic PCR assays and to improve the specificity of all the diagnostic techniques applied for the diagnosis of porcine spirochaetal colitis.

Finally, regarding the antimicrobial susceptibility of the identified “*B. hampsonii*” isolates, all of the tested isolates showed susceptibilities that were below the previously reported wild type cut-off values for the antimicrobial agents [42]. These results were not unexpected and are in accord with those obtained by Jansson et al. for different *Brachyspira* spp. isolated from wild-living mallards [12], likely due to the limited environmental exposure to antimicrobials among intestinal spirochaetes originating from wild birds. However, these values could be used as a baseline for monitoring the antimicrobial susceptibility of avian “*B. hampsonii*” isolates.

In conclusion, this study describes the presence of the newly described *Brachyspira* spp. “*B. hampsonii*” for the first time in waterfowl populations wintering in Northern Spain. This finding indicates that this spirochete is not limited to North America, and its presence in wild birds in Europe poses a risk of transmission to livestock. Consequently, additional studies are needed to determine the prevalence of “*B. hampsonii*” in the European swine population and the existence of other non-avian hosts for this *Brachyspira* species and the potential pathogenicity of “*B. hampsonii*” isolates recovered from waterfowl for swine.
